# Editorial: Adults with childhood onset disabilities: A lifespan approach

**DOI:** 10.3389/fneur.2022.1115869

**Published:** 2023-01-04

**Authors:** Elisabet Rodby-Bousquet, Mark D. Peterson

**Affiliations:** ^1^Faculty of Medicine, Lund University, Lund, Sweden; ^2^Centre for Clinical Research, Uppsala University, Uppsala, Sweden; ^3^Michigan Medicine, University of Michigan, Ann Arbor, MI, United States

**Keywords:** cerebral palsy [MeSH], adults [MeSH], health services for persons with disabilities [MeSH], spina bifida, health [MeSH], aging [MeSH]

## Introduction

Many individuals with a childhood onset disability (COD) such as cerebral palsy or spina bifida may experience near-normal life expectancies; however, healthcare coordination, clinical interventions, and research efforts remain largely focused on children with these conditions. For many individuals living with a COD, adult life comes with challenges in housing, employment, relationships, family life, and overall participation (Imms et al.; Pettersson and Rodby-Bousquet; Shrader et al.). The ability to participate in society without restrictions and a sense of belonging is rated as an important aspect of “meanings of citizenship” (van Heijningen et al.). This requires access to person-centered rehabilitation, social services and in many cases access to personal assistance (Pettersson and Rodby-Bousquet; van Heijningen et al.). Adults with CODs may experience higher risk for developing secondary conditions such as musculoskeletal morbidity (Dayanidhi; Won and Jung), pain (Jersak and Noritz; Jonsson et al.; Jacobson et al.), cardiometabolic diseases (Shin and Jung; Whitney et al.), kidney diesase (Whitney and Oliverio), dysphagia [Jonsson et al.; (1)], and mental health outcomes such as depression and anxiety (Jonsson et al.; Cohen et al.; Nguyen et al.) that may develop or be influenced by the disability, the presence of impairment, and/or the possibly accelerated process of aging. Consequently, there is a need for approaching health care delivery for adults with COD within the context of a life course health development model.

The goal of this Research Topic was to increase clinical and public health awareness of the unique healthcare needs facing adults living with cerebral palsy and other CODs, and to expand upon the social determinants of health (e.g., environmental, socioeconomic conditions, public policies, and etc.) that may contribute to disparities in or enhance the access, coordination, and continuity of clinical care for these populations.

Experts from multiple countries have documented a similar lack of clinical follow-up for individuals after they transition from pediatric to adult care, and inadequate longitudinal data to determine the natural history of CODs across a lifespan (Shrader et al.; Cornec et al.; Duncan et al.; Hurvitz et al.; Kingsnorth et al.; Ryan et al.; Starowicz et al.). Despite the increasing number of people living with CODs, there is also a lack of coordinated services and trained specialists and primary care providers who understand their unique medical needs, as these patients are still viewed collectively as having a “pediatric” medical condition. These problems exist at the level of the health care system, the clinician, and the individual (Hurvitz et al.). This is unquestionably troubling for youth with disabilities as they transition to adulthood and are left to navigate their confusing and disjointed options for continued healthcare services. The lack of coordinated care and access to services from adults with COD seems to be a global problem. In particular improved access to physiotherapy services for adults are urgently needed, and perceived as important to maintain physical activity and deal with the impact of aging on daily activities (van Heijningen et al.; Cornec et al.; Ryan et al.).

Sedentary behavior is increased in adults with cerebral palsy, particularly those with low functional capacities, as compared to adults without cerebral palsy, and they are less physically active than adolescents with cerebral palsy (Salie et al.). In fact, adults unable to change position independently are likely to spend around 23 h daily either in a sitting or lying position, and few use standing devices more than 1 h/day (Rodby-Bousquet and Agustsson). Finding strategies to keep adolescents and adults with CODs physically active and try to reduce sedentary behavior is important (Salie et al.).

Several clinical assessments, tests, and biomarkers are recommended for systematic follow-up of adults with COD to improve their care. These vary from cognitive assessments (Stadskleiv et al.), to screening for bone health (Won and Jung), body composition (Shin and Jung), pain (Jersak and Noritz), respiratory conditions (Jonsson et al.), kidney disease (Whitney and Oliverio), bladder and bowel function (Starowicz et al.), mental health (Cohen et al.; Nguyen et al.), physical activity and sedentary behavior (Salie et al.), asymmetric postures (Rodby-Bousquet and Agustsson), and IGF-1 involved in cognitive decline and dementia (Ng et al.).

Challenges with housing, employment, relationships, and participation in society for adults with CODs are apparent across countries and continents (Imms et al.; Pettersson and Rodby-Bousquet; Shrader et al.; van Heijningen et al.). Even though many adults with CODs have similar levels of education as the general population, they tend to have lower rates of employment and long term relationships (Shrader et al.). Better targeted societal resources and access to personal assistance for adults with CODs are urgently needed to allow equitable access to employment, independent living, enable full participation in society, and best possible outcomes in all aspects of life (Pettersson and Rodby-Bousquet; Shrader et al.; van Heijningen et al.).

In addition, adults with CODs face several barriers to health care at different levels, and have unique needs. The ability to initiate and coordinate their own care is sometimes complicated by challenges with cognitive function and communication (Hurvitz et al.; Stadskleiv et al.). They need continuity of care, and access to medical and health care professionals with expertise in these conditions and we need to develop evidence-based guidelines for health surveillance and provide appropriate interventions (van Heijningen et al.; Whitney et al.; Cornec et al.; Hurvitz et al.; Ryan et al.; Starowicz et al.). Thus, transition programs are desperately needed to bridge the gap from pediatric to adult care (Duncan et al.; Kingsnorth et al.).

## Conclusion

Despite the increasing number of adults living with CODs, there is a lack of coordinated services and adequately trained specialists to identify and treat preventable chronic conditions in these patients, as they are still viewed collectively as having a pediatric medical condition. Thought leadership is thus essential to increase research and promote best practices in preventive care, diagnosis, and disease management in these growing populations, as well as seamless transition and continuity of care as individuals with CODs grow up. This Research Topic is the first of its kind to assimilate research evidence regarding the unique healthcare needs facing individuals with CODs across the lifespan, and highlights the need for appropriate healthcare coordination and policies designed to enhance health and quality of life for all adults living with CODs.

## Author contributions

Both authors listed have made a substantial, direct, and intellectual contribution to the work and approved it for publication.
